# Inhärente Instabilität: zur Rolle der Output-Lücke im Stabilitäts- und Wachstumspakt

**DOI:** 10.1007/s10273-021-2876-7

**Published:** 2021-06-23

**Authors:** Christian M. Bender, Arne Heise

**Affiliations:** 1 Städtisches Kaufhaus, Universität Leipzig, Universitätsstraße 16, 04109 Leipzig, Deutschland; 2 Fakultät Wirtschafts- und Sozialwiss., Universität Hamburg, Welckerstraße 8, 20354 Hamburg, Deutschland

**Keywords:** H3, H63, H68

## Abstract

Fiskalregeln im Allgemeinen und der Stabilitäts- und Wachstumspakt im Speziellen werden wiederholt ins Feld geführt, um Staatsverschuldung effektiv zu begrenzen und fiskalische Nachhaltigkeit zu erzeugen. Besondere Aufmerksamkeit verdient hierbei das Konzept der unbeobachtbaren Output-Lücke.

## Abstract (en)

Fiscal rules in general and the Stability and Growth Pact in particular are repeatedly used to effectively limit public debt and achieve fiscal sustainability. In this context, the concept of the unobservable output gap deserves special attention.

Der Stabilitäts- und Wachstumspakt (SWP) ist ein regelbasiertes Konstrukt, das in seiner Historie mehrfache Änderungen und Reformen erfahren hat. Dabei sind nicht nur Richtwerte im Laufe der Zeit modifiziert, sondern auch dessen Bewertungsmaßstäbe und konzeptionellen Fundamente grundsätzlich revidiert worden. Um konjunkturelle Faktoren zu berücksichtigen, wurde seit 2011 auf den strukturellen Saldo abgestellt, der eine Konjunkturbereinigung vorsieht. Diese Konjunkturbereinigung fußt auf der Schätzung des unbeobachtbaren Potenzialoutputs, mit dessen Hilfe die Output-Lücke berechnet wird. Diese wird für die Berechnung des konjunkturellen Defizits verwendet, während im Rahmen der Ausgabenregel das Produktionspotenzial direkt eingeht. Die Produktionslücke dient somit indirekt über den strukturellen Saldo als Bewertungsmaßstab, als Entscheidungshilfe zur Einsetzung von Sanktionen und entscheidet darüber, ob Staaten einen nachhaltigen[1] fiskalpolitischen Ansatz verfolgen. Der strukturelle Saldo ist somit der allumfassende Bewertungsmaßstab einer regelgebundenen Fiskalpolitik in der Europäischen Union. Unter diesen Gegebenheiten dient ein Blick auf die Wirkmächtigkeit des strukturellen Saldos, der Output-Lücke und dessen Restriktivität.

## Methodologische Vorbetrachtungen

Während das Schuldenstandkriterium nach Maastricht einen Referenzwert von 60 % ausgibt, ist das korrespondierende Defizitkriterium im zeitlichen Verlauf wiederholt angepasst worden. Durch den Vertrag über Stabilität, Koordinierung und Steuerung in der Wirtschafts- und Währungsunion (SKS-Vertrag, auch Fiskalvertrag genannt) 2012 wurde letztlich ein Defizitwert formuliert, der um einmalige Ereignisse und konjunkturelle Einflüsse zu bereinigen ist. Das strukturelle Defizit ist hierbei auf 0,5 % gemessen am Bruttoinlandsprodukt (BIP) festgesetzt worden (Art. 3 SKS-Vertrag). Sofern ein Schuldenstand von 60 % oder niedriger erreicht wird, ist ein strukturelles Defizit von 1 % zulässig (Art. 3 SKS-Vertrag). Durch den SKS-Vertrag ist zudem die institutionelle Wirkungsrichtung erweitert worden. Während das traditionelle 3 %-Kriterium von der europäischen in die nationale Fiskalpolitik ausstrahlt, ist der SKS-Vertrag so angelegt, dass die einzelnen Vertragsbestandteile in nationales Recht mit Verfassungsrang überführt werden, wodurch die europäischen Vorgaben über die nationalen Verfassungen in die jeweilige Fiskalpolitik eingehen (Estella, 2018, 196). Die Fokussierung auf eine strukturelle Kenngröße stellt ein Novum in der gesamteuropäischen Fiskalpolitik dar und kann als politisch motivierte Zielgröße beschrieben werden. Die Höhe ist jedoch ökonomisch kaum begründbar, so dass sich die derzeitigen Richtwerte aus einem politischen Aushandlungsprozess herleiten lassen (De Grauwe, 2020, 127).[2] Der strukturelle Saldo wird durch ein aufwendiges ökonomisches Prozedere ermittelt (vgl. Tabelle 1).

1 Die Nachhaltigkeit wird über die Mittelfristigen Haushaltsziele (MTO) definiert. Auf das Konzept wird im weiteren Verlauf eingegangen.

2 Die 3 %-Defizitgrenze folgt aus der Domar'schen Finanzarithmetik, wenn man ein langfristiges Nominalwachstum des BIP der Euroländer von 5 % und eine zu stabilisierende Schuldenstandsquote von 60 % unterstellt. Somit bezieht sich der Hinweis auf den politischen Aushandlungsprozess in erster Linie auf die arbiträr gesetzte Schuldenstandsquote.

**Christian Bender** ist wissenschaftlicher Mitarbeiter am Lehrstuhl für Finanzwissenschaften an der Universität Leipzig.

**Prof. Dr. Arne Heise** ist Lehrstuhlinhaber für Finanzwissenschaft und Public Governance an der Universität Hamburg.

Er stellt einen zyklisch angepassten Saldo dar, von dem temporäre Abweichungen abgezogen werden (Havik et al., 2014). Der zyklisch angepasste Saldo selbst ist ein Haushaltssaldo in Abhängigkeit zum BIP, von dem wiederum die zyklische Komponente der Output-Lücke abgezogen wird. Die zyklische Anpassung erfolgt über die Budgetsemielastizität, während die Output-Lücke auf dem Konzept des Potenzialoutputs beruht (Ademmer et al., 2019; Lenk et al., 2020a). Das Potenzialoutput wird über eine Cobb-Douglas-Produktionsfunktion bestimmt, bestehend aus den Inputfaktoren Arbeit und Kapital, wie auch einem Produktivitätsparameter. Die jeweiligen Bestandteile der Inputfaktoren werden zyklisch bereinigt und gehen anschließend in die Berechnung ein.

Folglich zeigt sich, dass das zugrunde liegende Konzept des Potenzialoutputs und somit die Output-Lücke revisionsanfällig[3] sein können. Die Bestimmung basierend auf einem Produktionsfunktionsansatz ist keine beobachtbare, sondern eine errechnete Größe. Dies impliziert letztlich, dass potenzielle Revisionen einen großen Einfluss auf die Schlüsselvariable haben können, die für die europäische Fiskalpolitik einen hohen Stellenwert hat.

### Mittelfristiges Haushaltsziel (MTO) als Bewertungskriterium

Wird der strukturelle Saldo als Bewertungsmaßstab herangezogen, so zeigt sich eine große Heterogenität im zeitlichen Verlauf (vgl. Abbildung 1). Dabei wird deutlich, dass sich Phasen der fiskalischen Expansion und Austerität für die einzelnen Länder unterschiedlich stark ausprägen. Positive Salden werden nur durch Deutschland und die Niederlande erreicht, wie auch durch Griechenland, das im Rahmen der Hilfsprogramme ab 2010 umfassende fiskalische Anpassungsprogramme zu durchlaufen hatte.

Hingegen konnten Spanien und Frankreich die Zielsetzung eines strukturellen Saldos von 0,5 % nicht erreichen. Trotz der großen Heterogenität im Hinblick auf die Zielerreichung zeigt die euroländerweite Entwicklung – insbesondere im Vergleich zu den USA[4] – eine starke Restriktionswirkung des europäischen Governance-Systems in Richtung der 0,5 %-Zielmarke für den strukturellen Saldo.

Tabelle 1
**Ermittlung des strukturellen Saldos (EU-Konzept)**

| | Formale Beschreibung | Bezeichnung |
|---|---|---|
| (1) | $SB_t = CAB_t - OE_t$ | struktureller Saldo (aggregiert) |
| (2) | $SB_t = (BAL / Y)_t - \varepsilon OG_t - OE_t$ | struktureller Saldo |
| (3) | $OG_t = Y_t / PO_t - 1$ | relative Output-Lücke |
| (4) | $PO_t = K^{\alpha} L^{1-\alpha} \Gamma_t$ | Potenzialoutput |
| (5) | $L = POPW * PARTS * (1 - NAWRU) * HOURS$ | Inputfaktor Arbeit |
| (6) | $K = I + (1 - dep)\, K(-1)$ | Inputfaktor Kapital |

SB = struktureller Saldo; CAB = zyklisch angepasster Saldo; OE = one-off Messungen und temporäre Abweichungen; BAL = Haushaltssaldo; $\varepsilon$ = Budgetsemielastizität; OG = Output-Lücke; Y = BIP; PO = Potenzialoutput; K = Kapital; L = Arbeit; $\Gamma$ = Produktivitätsfaktor; POPW = Bevölkerung im erwerbsfähigen Alter; PARTS = geglättete Partizipationsrate; HOURS = durchschnittliche pro Kopf geleistete Arbeitsstunden; dep = Abschreibungsrate; NAWRU= den Lohnanstieg nicht beschleunigende Arbeitslosenquote.

Quelle: Lenk et al. (2020a, 6).

Bedingt durch die Corona-Pandemie erscheint es jedoch unumgänglich, die bestehenden Regelungen für besondere Krisensituationen zu nutzen. Die Europäische Kommission hat folgerichtig in einer Erklärung den generellen Ausnahmetatbestand im Zuge des SWP ausgerufen (Europäische Kommission, 2020).

Angesichts des Beitrags zur Zielerreichung in der Vor-Corona-Zeit ist somit nicht nur von Relevanz, ob die Referenzwerte erreicht wurden, sondern auch, ob das korrespondierende mittelfristige Haushaltsziel (MTO) als operatives fiskalisches Ziel eingehalten werden konnte, das zur Erreichung der übergeordneten Fiskalregeln gilt. Das MTO stellt einen länderspezifischen Referenzwert dar, der über drei Jahre hinweg zu erreichen ist. Das MTO ist hierbei struktureller Natur, um einen direkten Vergleich mit dem strukturellen Saldo gewährleisten zu können. Das MTO setzt sich aus drei Komponenten zusammen (vgl. Tabelle 2).

Komponente (1) ($MTO^{SA}$) dient als Sicherheitsabstand zum 3 %-Maastricht-Defizitkriterium. Dieses setzt sich aus einem einfachen Durchschnitt zwischen der länderspezifischen Standardabweichung der zyklischen Komponente des Budgetsaldos und der Standardabweichung der zyklischen Komponente des Budgetsaldos aller Mitgliedsländer seit 1985 zusammen. Hierbei werden sukzessive negative Output-Lücken durch ein 25-jähriges rollendes Zeitfenster in die Betrachtung aufgenommen (Europäische Kommission, 2019, 10 f.). Anschließend erfolgt eine Multiplikation mit einem Gewichtungsfaktor von 1,2, der sich auf den durchschnittlichen Sicherheitsabstand gemessen am BIP aller 27 Mitgliedstaaten bezieht. Zudem ist durch eine Nebenbedingung ein Varianzfenster

3 Revisionen stellen Korrekturen des Datenbestands dar. Diese können durch unterschiedliche Gründe hervorgerufen werden. Z. B. können Veränderungen der Berechnungsgrundlage, Datenänderungen oder das Hinzukommen neuer Datenpunkte zu Revisionen führen.

4 Um die Einheitlichkeit der Datengrundlage sicherzustellen, wurde auf die Datenbank AMECO zurückgegriffen. Da diese keine Daten zum strukturellen Defizit der USA ausweist, sind die USA nicht in die Betrachtung in Abbildung 2 einbezogen. Andere Datenquellen (IWF) zeigen aber, dass das strukturelle Defizit der USA nur 2014 und 2015 geringer ausfiel als -3,0 % und ansonsten zwischen -8,1 % (2010) und -6,8 % (2019) mit einem Jahresdurchschnitt von -4,8 % zwischen 2010 und 2019 lag.

Abbildung 1
**Strukturelle Salden in ausgewählten Euro-Ländern**

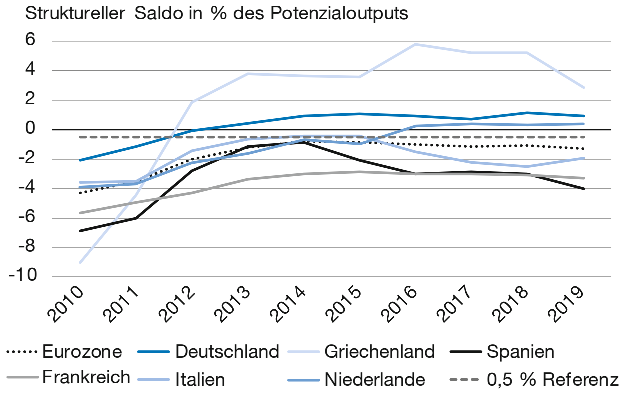

Anmerkung: Datengrundlage bildet die Annual Macro-Economic Database (AMECO) der EU. Im Sample sind nord- und südeuropäische Länder enthalten, die unterschiedlich stark von den ökonomischen Verwerfungen nach 2008 getroffen wurden.

Quelle: AMECO (2020); eigene Darstellung.

geschaffen worden, das dabei helfen soll, exzessiv strenge oder nachsichtige Sicherheitsabstände zu vermeiden.

Für jeden Mitgliedstaat wird zudem ein Nachhaltigkeitsindikator über Komponente (2) ($MTO^{NsF}$) aufgenommen, der sich auf den Stand der Verschuldung bezieht und eine Konvergenz zu umsichtigen Verschuldungsniveaus garantieren soll. Diese setzt sich aus einem Budgetgleichgewicht zusammen, das die Schuldenstandquote auf 60 % stabilisieren würde (Europäische Kommission, 2019, 12 f.). $\alpha A$ repräsentiert die budgetäre Anpassung, die einen Anteil der derzeitigen Alterungskosten abdeckt, wobei die Kommission einen $\alpha$-Wert von 33 % ansetzt (Europäische Kommission, 2019, 12). $X$ beschreibt eine ergänzende Verschuldungsreduktion, sofern die Schuldenstandquote über 60 % liegt.

Mitglieder der Eurozone müssen zudem eine untere Grenze von -1 % gemessen am BIP über Komponente (3) ($MTO^{Euro}$) einhalten. Alle drei Komponenten werden anschließend in eine Maximierungsfunktion ($MTO^{MIN}$) überführt, und es wird eine länderspezifische Untergrenze ermittelt, die mit dem geringstmöglichen MTO korrespondiert, das alle Kriterien erfüllt (Europäische Kommission, 2019, 12).

## Abweichung vom Soll: die Anpassungsmatrix

Die Komponenten zur Ermittlung des MTO zeigen, dass auch hier das Konzept des Potenzialoutputs verwendet wird, um die Output-Lücken zu berechnen. Die Probleme, die sich im Zuge der Konjunkturbereinigung und der Revisionsanfälligkeit für den strukturellen Saldo selbst ergeben, liegen somit auch für dessen Bewertungsmaßstab, das MTO, vor. Mögliche Verzerrungen wirken jedoch

Tabelle 2
**Komponenten des MTO**

| | Formale Beschreibung | Bezeichnung |
|---|---|---|
| (1) | $-3 + 1{,}2\,\frac{[stdev_i\,(\varepsilon_i * OG_i) + stdev_{EU}\,(\varepsilon_{EU} * OG_{EU})]}{2}$ <br> s. t. $-0{,}7 \geq MTO^{SA} \geq -1{,}5$ | $MTO^{SA}$ |
| (2) | $GG + \alpha A + X;\ X = 0{,}024 \cdot \omega - 1{,}24$ | $MTO^{NsF}$ |
| (3) | -1 % | $MTO^{Euro}$ |
| (4) | max ($MTO^{SA}$; $MTO^{NsF}$; $MTO^{Euro}$) | $MTO^{MIN}$ |

Stdev=Standardabweichung; $\varepsilon_i$ = Budgetsemielastizität; OG = Output-Lücke; Index EU = Aggregat aller Mitgliedsstaaten der EU seit 1985; $GG$ = Budgetäres Gleichgewicht; $\alpha$ = Gewichtungsfaktor der Alterungskosten; $A$ = Budgetäre Anpassung an die Alterung der Gesellschaft; $X$ = Verschuldungsreduktion; $\omega$ = Schuldenstandquote in % vom BIP.

Quelle: Europäische Kommission (2019, 11 ff.); eigene Darstellung.

asymmetrisch. Während für den strukturellen Saldo keine Nebenbedingung angegeben wird, liegt für das MTO eine implizite Restringierung vor, da über die erste Komponente eine Begrenzung der Varianz vorgenommen wird. Dennoch lässt sich im begrenzten Maße feststellen, dass sich Instabilitäten, ausgelöst durch das Design der Output-Lücke, auf das MTO, wie auch den strukturellen Saldo auswirken.

Der Vergleich beider Kenngrößen in Abbildung 2 zeigt, dass nur Deutschland und die Niederlande ihre MTO einhalten konnten. Für Griechenland gibt es aufgrund der anhaltenden ökonomischen Anpassungsprogramme kein MTO. Hingegen verfehlen Spanien, Frankreich und Italien ihre MTO 2019. Dieser Befund dürfte sich im Hinblick auf die Corona-Pandemie und die korrespondierenden fiskalischen Auswirkungen nochmals verschärft haben. Es zeigt sich jedoch eine geringere Fluktuation der einzelnen MTO im Vergleich zu den Strukturellen Salden.

Sofern ein Mitgliedstaat sein MTO nicht erreicht hat, ist ein Anpassungspfad zu etablieren, der jährliche Verbesserungen vorsieht, wobei für Euro-Länder ein Benchmark von 0,5 % gemessen am BIP vorliegt (Europäische Kommission, 2019, 15). Eine Übersicht für die jeweiligen Anpassungspfade gibt Tabelle 3. Die fiskalische Anstrengung variiert je nach ökonomischem Zyklus und wird anhand der Output-Lücke gemessen. Je größer (kleiner) die Output-Lücke ist, desto größer (kleiner) ist die verlangte Anpassung.[5] Hierbei gilt, dass der verlangte Anpassungspfad zum MTO für das Jahr *t* im Frühjahr *t*-1 eingefroren wird, um die Prognosequalität zu verbessern. Auch hier kommt das Potenzialoutput zum Einsatz, indem der Anpassungspfad durch die Output-Lückenbedingungen determiniert wird.

5 Die Output-Lücke wird anteilig am Potenzialoutput angegeben, wobei eine negative (positive) Output-Lücke vorliegt, wenn das derzeitige BIP unterhalb (oberhalb) des Potenzials liegt.

Abbildung 2
**Vergleich von MTO und strukturellem Saldo**

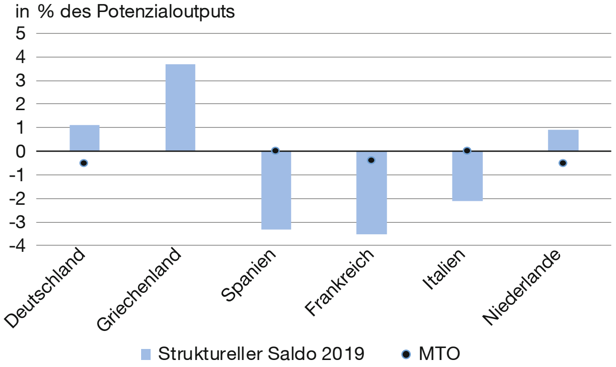

Quelle: DG ECFIN (2020); eigene Berechnungen; eigene Darstellung.

Tabelle 3
**Anpassungspfad bei Nichterreichung des MTO**

| Position | Verlangte jährliche Anpassung (%-Punkte gemessen am BIP) | | |
|---|---|---|---|
| | Bedingung | Verschuldung ≤ 60 % und geringe/mittlere Nachhaltigkeitsrisiken | Verschuldung > 60 % oder hohe Nachhaltigkeitsrisiken |
| Außergewöhnlich schlecht | Reales Wachstum < 0 oder OG < -4 | Keine Anpassung benötigt | |
| Sehr schlecht | -4 ≤ OG ≤ -3 | 0 | 0,25 |
| Schlecht | -3 ≤ OG ≤ -1,5 | 0 falls Wachstum unter Potenzial | 0,25 falls Wachstum unter Potenzial |
| | | 0,25 falls Wachstum über Potenzial | 0,5 falls Wachstum über Potenzial |
| Normal | -1,5 ≤ OG ≤ 1,5 | 0,5 | > 0,5 |
| Gut | OG ≥ 1,5 | > 0,5 falls Wachstum unter Potenzial | ≥ 0,75 falls Wachstum unter Potenzial |
| | | ≥ 0,75 falls Wachstum über Potenzial | ≥ 1 falls Wachstum über Potenzial |

Quelle: Europäische Kommission (2019, 17); eigene Darstellung.

Somit lässt sich festhalten, dass das Konzept des Potenzialoutputs bei der Ermittlung des strukturellen Saldos (Vergleichsvariable), dem Bewertungsmaßstab (MTO) und dem Anpassungspfad bei Abweichung von MTO (Anpassungsmatrix) zum Zuge kommt. Mögliche Instabilitäten wirken demnach auf alle drei Prozesse mit unterschiedlicher Intensität auf die Fiskalpolitik ein. Während im Zuge des strukturellen Saldos eine weitere Gewichtung durch die Budgetsemielastizität vorgenommen wird, wird im Hinblick auf das MTO eine Varianzbegrenzung durch die Nebenbedingung im Zuge der Berechnung des Sicherheitsabstands vorgenommen. Hinsichtlich der Bewertung des Anpassungspfads bei Abweichung vom MTO wird hingegen die Output-Lücke in unmodifizierter Form weiterverwendet.

## Zur Krux von Revisionen der Output-Lücke

Um aufzuzeigen, in welchem Ausmaß Instabilitäten auf die drei beschriebenen Transmissionskanäle einwirken, werden die Revisionen der Output-Lücke betrachtet.[6] Revisionen können z. B. aufgrund einer verbesserten Datenbasis oder Veränderungen der Berechnungsgrundlage auftreten. Insbesondere letzteres verweist auf die problematische Natur einer Politik- und Kontrollvariable, die keinen eindeutigen Instrumentencharakter hat: Der mithilfe der Output-Lücke berechnete strukturelle Saldo dient zwar als Zielgröße der Finanzpolitik, ist aber vom politischen Akteur nur bedingt steuerbar. Sie hängt bei unterstellter Konstanz der Budgetsemielastizität von einer erwarteten Größe, der erwarteten Output-Lücke, ab. Jede Fehlerwartung – aufgrund exogener Schocks oder unerwarteter endogener Änderungen – führt zu einer Revision des strukturellen Saldos und folglich einer Neubewertung des finanzpolitischen Kurses.

Abbildung 3 zeigt die Output-Lücken aus zwei Zeitpunkten an. Hierbei wird einerseits diese zum Zeitpunkt *t* (z. B. Output-Lücke aus 2010 für 2010) mit jener zum Zeitpunkt *t*+1 (z. B. Output-Lücke aus 2011 für 2010) verglichen. Stimmen beide Werte überein, ergibt sich eine 45°-Linie. Liegen Revisionen vor, weichen beide Werte voneinander ab, wodurch sich Abweichungen von der Geraden ergeben. Wie die Abbildung zeigt, existieren signifikante Abweichungen, wodurch geschlussfolgert werden kann, dass Revisionen einen beträchtlichen Einfluss auf die Output-Lücke haben.

Um aufzuzeigen, wie groß diese Position ausfallen kann, wird durch die Abbildungen 4 und 5 dargestellt, wie sich die einzelnen Revisionen für Deutschland und Griechenland verhalten. Dabei werden die einzelnen Korrekturen für jedes Jahr aufsummiert, um zu zeigen, welcher Volatilität die Berechnung der Output-Lücke im zeitlichen Ablauf ausgesetzt ist. Für 2011 ergibt sich somit insgesamt ein Revisionsvolumen von 4,5 Prozentpunkten. Der Betrag setzt sich durch eine Revision nach unten in Höhe von -0,4 Prozentpunkten (von 2017 gegenüber 2016) und andernfalls durch positive Revisionen zusammen.[7] Die Aufsummierung aller Revisionszeitpunkte ergibt das absolute Revisionsvolumen für 2011 über alle Berichtszeiträume hinweg. Bis auf den derzeitigen Prognoserand in 2020 lässt sich erkennen, dass positive Revisionen überwiegen. Dies impliziert, dass im Jahr *t*+1 eine tendenziell höhere Output-Lücke ausge-

6 Die Output-Lücken wurden jeweils den Frühlingsprognosen der Generaldirektion Wirtschaft und Finanzen entnommen. Hierbei wurde explizit auf die Frühlingsprognosen abgestellt, um eine erste Betrachtung für 2020 aufnehmen zu können.

7 Für jedes Jahr von 2010 bis 2019 wurden die entsprechenden Differenzen der Zeitfenster gebildet und betragsmäßig aufsummiert, um das Revisionsvolumen zu bilden.

Abbildung 3
**Streudiagramm der Output-Lücke**

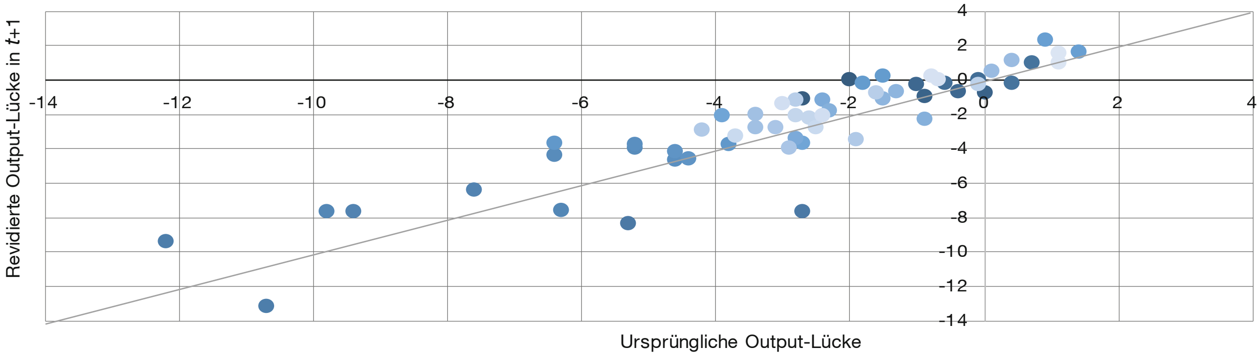

Quelle: Frühlingsprognosen der DG ECFIN (2020); eigene Berechnungen; eigene Darstellung.

wiesen wird als im Jahr *t*. Durch höhere positive Output-Lücken wird somit unterstellt, dass die wirtschaftliche Auslastung der Produktionsfaktoren wesentlich umfangreicher stattgefunden hat, als zuvor angenommen wurde – die konjunkturelle Entwicklung war also besser als erwartet. Neben der Zunahme der Intensität positiver Output-Lücken gehen auch jene positiven Veränderungen ein, die zu einer Abnahme bestehender negativer Output-Lücken führen. Anders verhält es sich z. B. in 2020, wobei durch die Frühjahrsprognose vom April 2020 eine erste Einschätzung vorliegt. Während in 2019 eine Output-Lücke für 2020 von -0,1 % gemessen am Potenzialoutput geschätzt wurde, liegt die derzeitige Prognose für 2020 bei -6,4 %.

Für Griechenland verhält sich die Sachlage im Vergleich zu Deutschland anders. Wie ersichtlich wird, ist der überwiegende Anteil der Revisionen negativer Natur, was impliziert, dass ex ante die wirtschaftliche Situation besser prognostiziert wurde, als jene, die sich bei einer nachträglichen Betrachtung ergab (Hauptmeier und Leiner-Killinger, 2020). Die positiven Erwartungen, die prognostiziert werden, stellen sich somit nicht ein. Bemerkenswert ist, dass insgesamt das Revisionsvolumen z. B. in 2010 und 2011 um ein Vielfaches über dem Revisionsvolumen für Deutschland liegt. So beträgt das Verhältnis allein 2010 zirka 1:7. Letztlich muss konstatiert werden, dass im weiteren Zeitverlauf noch Revisionszeitpunkte durch weitere Prognosen der Kommission ausstehen. So ist davon auszugehen, dass gerade für den aktuellen Datenrand durch die nächsten Prognosen noch weitere Revisionen hinzukommen, die das Revisionsvolumen maßgeblich beeinflussen können.

Um eine Einschätzung für die gesamte Stichprobe geben zu können, zeigt Abbildung 6 das gesamte Revisionsvolumen aller betrachteten Euro-Länder. Hierbei wird deutlich, dass eine massive Heterogenität zwischen den einzelnen Ländern von 2010 bis 2012 vorliegt, die sich erst im weiteren Zeitverlauf maßgeblich reduziert. Ob dies eine direkte Konsequenz aus den Anpassungen des SWP ist und somit eine qualitative Verbesserung der Berechnungsgrundlage darstellt, oder ob dies auf die geringere Zahl an möglichen Revisionszeitpunkten zurückzuführen ist, kann nur ansatzweise beantwortet werden.

Lenk et al. (2020b, 379) kommen zu dem Ergebnis, dass in Bezug auf Deutschland die Modifikationen des europäischen Konzepts zur Ermittlung der Output-Lücken bisher nicht dazu beitragen konnten, die Revisionsvolumina maßgeblich zu reduzieren. Zudem fehlt eine klar definierte Zielgröße. Zwar existiert ein Plausibilitätstool der Europäischen Kommission bei paradox erscheinenden Outputlückenberechnungen, jedoch unterliegen auch diese schlussendlich der Bewertung durch die Kommission, die aufgrund von Erfahrungswerten vorgenommen wird (Europäische Kommission 2019, 19 f.). Die Arbeitsgruppe für Output-Lücken trifft sich viermal im Jahr auf europäischer Ebene, um die Berechnungsgrundlagen und Resultate zu besprechen und gegebenenfalls zu modifizieren (Huber et al. 2017, 5). Wie die Daten jedoch zeigen, können die Revisionen nicht einheitlich für alle Länder maßgeblich am Datenrand, für die bereits mehrere Revisionszeitpunkte vorliegen, reduziert werden. Ökonometrische Verfahren scheinen somit einen begrenzten Einfluss auf die Güte der Output-Lücken-Berechnungen zu haben.

## Auswirkungen der Revisionen auf die Transmissionskanäle

Es stellt sich die Frage, welche Auswirkungen die Revisionen der Output-Lücke auf den strukturellen Saldo, das MTO und die Bewertungsmatrix haben. Erhöht sich die positive Output-Lücke bedingt z. B. durch eine Unterschätzung der konjunkturellen Dynamik, so verringert sich bei gleichbleibender Budgetsemielastizität der zyklisch angepasste Saldo, wodurch letztlich der strukturelle Saldo

Abbildung 4

**Revisionen der Output-Lücke für Deutschland von 2010 bis 2020**

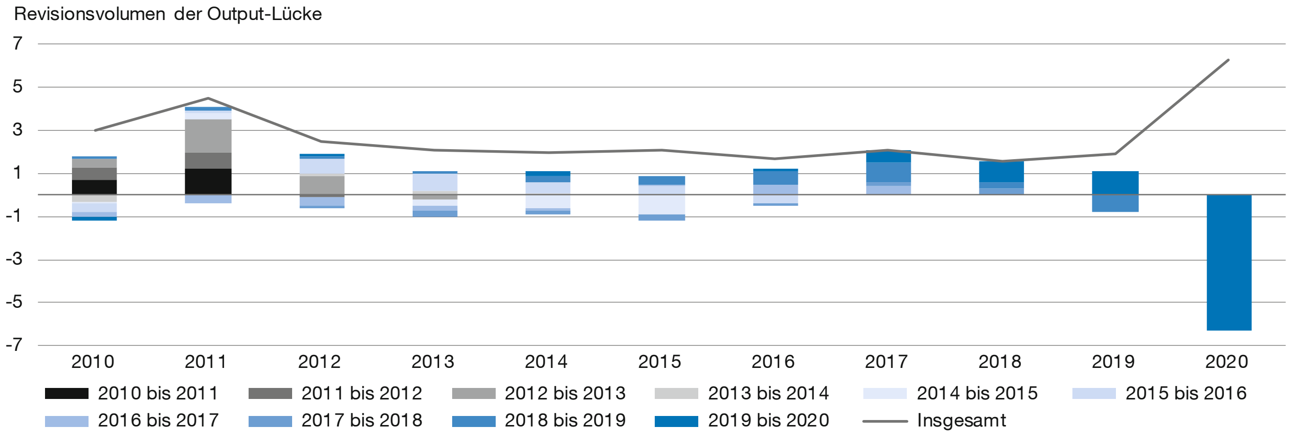

Quelle: Frühlingsprognosen der DG ECFIN (2020); eigene Berechnungen; eigene Darstellung.

Abbildung 5

**Revisionen der Output-Lücke für Griechenland von 2010 bis 2020**

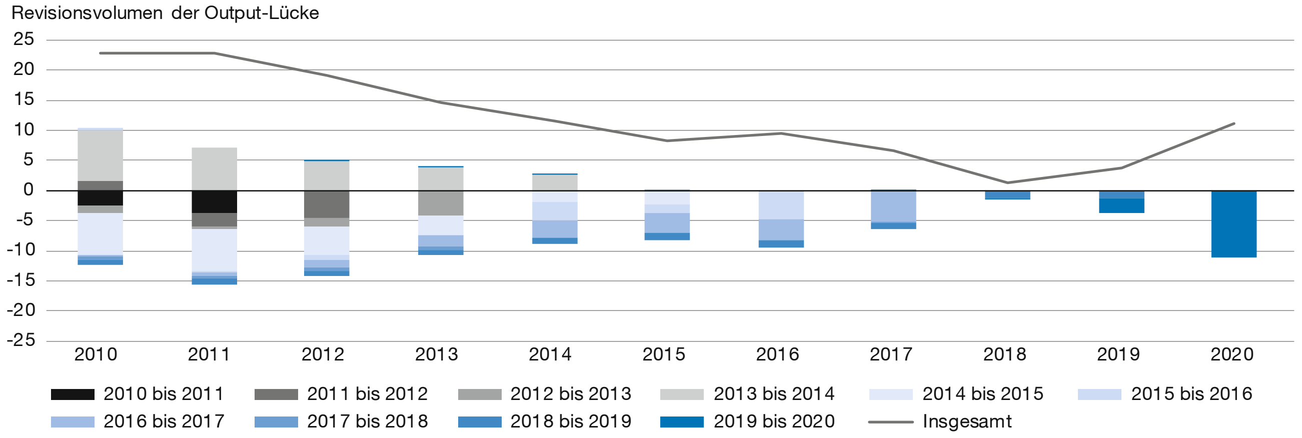

Quelle: Frühlingsprognosen der DG ECFIN (2020); eigene Berechnungen; eigene Darstellung.

reduziert wird und vice versa führt eine Verringerung der positiven Output-Lücke zu einem Anstieg des strukturellen Saldos (Lenk et al. 2020b, 378). Im Falle einer Erhöhung der negativen Output-Lücke z. B. aufgrund zu optimistischer Erwartungen in $t_0$ wird sich das strukturelle Defizit auch erhöhen. Da die temporären Abweichungen und die Budgetsemielastizität unabhängig von der Output-Lücke ermittelt werden, erfolgt somit keine weiterführende Revisionsangleichung. Die Revisionen wirken vollumfänglich auf den strukturellen Saldo ein. Dies führt jedoch nicht zu einer Neubewertung der fiskalischen Situation ex post. Somit sind die mit dem strukturellen Saldo verbundenen wirtschaftspolitischen Maßnahmen nicht mehr reversibel. Auf diese Weise wird der strukturelle Saldo ohne wirtschaftspolitische Maßnahmen gesenkt oder erhöht. Anders ausgedrückt kann eine fehlerhafte Bewertung des strukturellen Saldos zu nicht wünschenswerten fiskalischen Effekten führen. Die Revisionszusammensetzungen für die Output-Lücken zeigen, dass die Mehrzahl an Revisionen für Deutschland, Frankreich und die Niederlande positiv ist, während für Griechenland, Spanien und Italien zumeist negative Revisionen vorliegen. Speziell die südeuropäischen Staaten scheinen somit die positive Erwartungshaltung, die bei der Berechnung der Output-Lücken unterstellt wurde, nicht erfüllt zu haben. Sie erfahren nun einen Konsolidierungsdruck, der nicht in einer unsoliden Gestaltung der Finanzpolitik, sondern in unerfüllten Erwartungen begründet ist. Spiegelbildlich verhält es sich für die nordeuropäischen Staaten, die durch eine relative Besserstellung bei Errechnung des strukturellen Saldos erkennen, dass sie vorhandene Handlungsspielräume ungenutzt ließen. Wenn man, wie es z. B. für Deutschland zwischen 2010 und 2020 der Fall war, in den meisten Jahren die Situation überschätzter negativer Output-Lücken

Abbildung 6

**Kumuliertes Revisionsvolumen aller Euro-Länder im Vergleich**

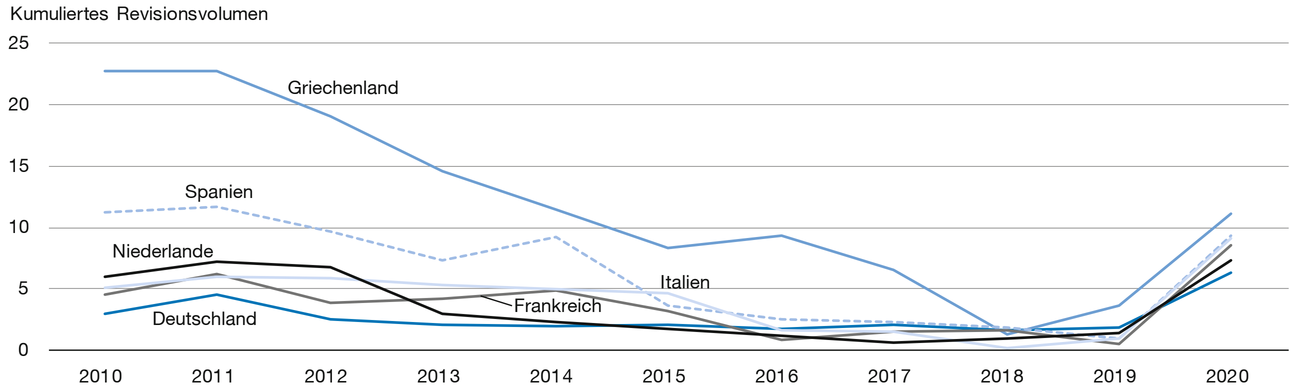

Quelle: Frühlingsprognosen der DG ECFIN (2020); eigene Berechnungen; eigene Darstellung.

unterstellt und die übermäßig restriktive Finanzpolitik Deutschlands (gemessen am Referenzwert von -0,5 %, und im Vergleich zum EA-Durchschnitt; vgl. Abbildung 1) in Rechnung stellt, werden die Hintergründe für den öffentlichen Investitionsstau nachvollziehbar (z. B. Krebs und Scheffel, 2017).

Die bessere Einschätzung der Situation ex ante hält letztlich Schlussfolgerungen für die MTO bereit. Durch die Varianzbegrenzung, bedingt durch die Nebenbedingung der MTO, zeigt sich, dass Komponente (1) aus Tabelle 2 nur eine begrenzte Restriktivität aufweist, da der Varianzparameterraum eingeschränkt wird. Der Instabilitätskanal wird folglich über das Nachhaltigkeitskriterium erzeugt, indem die Verschuldung von über 60 % gemessen am BIP durch das MTO gefordert wird. Dessen Umfang ist direkt mit dem Verschuldungsgrad verknüpft.[8] Somit ist es nicht direkt die Revision, die über die Output-Lücken auf das MTO massiv einwirkt, sondern vielmehr die Schuldenstandquote selbst. Gebündelt mit der Fehlbewertung des strukturellen Saldos durch die Materialisierung von Revisionen kann so eine fehlerhafte Verflechtung von Anforderung und Status quo entstehen, die sich unter Umständen negativ auf das fiskalpolitische Gefüge der Staaten auswirkt.

Dies wird zusätzlich durch die Bewertungsmatrix für den Anpassungspfad hin zum MTO unterstützt (vgl. Tabelle 3). Hier wirken die Output-Lücken ohne Begrenzung direkt auf den Anpassungspfad ein. Diese werden ausschließlich durch einen Nachhaltigkeitsindikator und die Schuldenstandquote flankiert, wobei letztere ebenfalls kritikwürdig erscheint. Alle Staaten in der Betrachtung weisen eine Verschuldung von über 60 % gemessen am BIP aus, wobei die Output-Lücken für 2020 bereits als außergewöhnlich schlechte Position nach der Bewertungsmatrix einzuschätzen sind. Hauptmeier und Leiner-Killinger (2020, 297) zeigen, dass die Granularität der Bewertungsmatrix zudem zu einer möglichen Fehleinordnung der Staaten führt, wodurch eine prozyklische Fiskalpolitik unterstützt werden könnte. Setzt sich jedoch die vorherrschende Revisionsdynamik fort, so wird gerade für Deutschland, Frankreich und die Niederlande der Konsolidierungsdruck zukünftig zunehmen, da positive Revisionen am ehesten zu erwarten sind.

8 Die zweite Frage ist, ob das 60 % Defizitkriterium nachhaltig ist. Dessen Entstehungsgeschichte lässt vermuten, dass es sich um einen statischen Wert ohne ökonomische Begründung handelt (Lenk et al., 2020c).

## Reformdruck und bedingte Möglichkeiten

Es wurde deutlich, dass der Output-Lücken-Berechnung, der die Potenzialoutputschätzungen zugrunde liegen, eine gewichtige Dynamik innewohnt. So können einzelne Modifikationen nicht nur Auswirkungen für den strukturellen Saldo, sondern auch für die MTO und die zukünftigen Bewertungen über den präventiven Arm des SWP haben, woraus sich zusätzlicher Konsolidierungsdruck ableiten lässt.

Änderungen der derzeitigen Methodik lassen sich nicht auf nationaler Ebene ausmachen, da sich die Mitgliedstaaten zur Implementierung der EU-Methodik über den SKS-Vertrag verpflichtet haben. Vielmehr ist die europäische Ebene gefragt. Einerseits kann dies über die technische Ebene erfolgen. Hierzu sind Änderungen notwendig, die durch das Economic Policy Committee (EPC) empfohlen werden. Dieses Komitee unterstützt die Arbeiten der Kommission und des Rates im ECOFIN-Format. Zudem existiert die Ouput Gaps Working Group, eine Arbeitsgruppe angesiedelt im EPC, die sich fortlaufend mit der Evaluierung der Output-Lücken beschäftigt.

Auf technischer Ebene ist über ein Bottom-up-Verfahren eine Veränderung der Gegebenheiten möglich. Alternativ ist auch ein Top-down-Prozess gangbar, indem der ECOFIN-

Rat um Änderungen der Berechnungsmethodik bittet, und so durch die politische Ebene ein Reformvorgang angestoßen wird. Dieser Vorgang ist durchaus intrinsisch motiviert. Da die Revisionsanfälligkeit den strukturellen Saldo, das MTO und den Bewertungsmaßstab gleichermaßen betrifft, ist eine relative Besserstellung durch eine Modifikation der Berechnung möglich, die zu weniger revisionsanfälligen Ergebnissen führt. Hinsichtlich des strukturellen Saldos ist diese Betrachtungsweise von Relevanz, sofern sich hierdurch der fiskalische Handlungsspielraum einzelner Mitgliedstaaten erhöhen sollte.
